# Multidrug-Resistant *Escherichia coli* Bacteremia

**DOI:** 10.3201/eid1910.130309

**Published:** 2013-10

**Authors:** Fahad Alhashash, Vivienne Weston, Mathew Diggle, Alan McNally

**Affiliations:** Nottingham Trent University, Nottingham, United Kingdom (F. Alhashash, A. McNally);; Nottingham University Hospitals National Health Service Trust, Nottingham (V. Weston, M. Diggle)

**Keywords:** ExPEC, MLST, ESBL, antibiotic resistance, antimicrobial resistance, bacteremia, E. coli, Escherichia coli, bacteria, multidrug resistance

**To the Editor:** Extraintestinal pathogenic *Escherichai coli* (ExPEC) bacteria have the ability to cause diverse and serious diseases, such as urinary tract infections (UTIs) and bacteremia (*1*–*3*); incidence of bacteremia is increasing globally (*4*). The emergence of multidrug resistance in *E. coli* is also becoming a global concern, with particular emphasis on *E. coli* sequence type (ST) 131, which is being increasingly reported in UTIs. Drug resistance is mediated by extended-spectrum β-lactamases (ESBLs), mainly of the CTX-M family, particularly CTX-M-15 and 14, and less frequently of the SHV and OXA families (*5*,*6*). Few studies are available regarding the characterization of *E. coli* strains causing bacteremia.

We characterized 140 *E. coli* isolates from bacteremia patients treated at Nottingham University Hospital (Nottingham, UK) over a 5-month period, with the aim of developing an epidemiologic profile of the population of ExPEC that causes bacteremia. For context, we compared the isolates with 125 *E. coli* isolates from urine samples collected during the same period. Cases were selected to include isolates from a diverse patient group: patient ages ranged from 1 month to 90 years; patient sex was evenly divided between male and female; infections were community- and hospital-associated; and suspected sources of infection varied. Antimicrobial drug susceptibility tests, PCR detection of ESBL genes_,_ multilocus sequence typing using the Achtman scheme (http://mlst.ucc.ie/mlst/dbs/Ecoli), and virulence-associated gene (VAG) carriage screening by PCR were performed on isolates as described (*7*).

Significantly more bacteremia *E. coli* isolates than urine *E. coli* isolates were resistant to ciprofloxacin (25.7% vs. 8.8%; p<0.001) and cefradine (20.0% vs. 11.2%; p<0.05). These results were reflected in the number of isolates in the 2 populations displaying a multidrug- resistance phenotype (resistance to antimicrobial drugs belonging to >2 classes); a significantly higher number of multidrug-resistant bacteremia *E. coli* isolates than multidrug-resistant urine isolates were found (50.7% vs. 32%; p = 0.01). PCR screening for ESBL carriage showed significantly higher ESBL carriage in bacteremia *E. coli* isolates than urine isolates for *bla*_SHV_ (15.7% vs. 5.6%; p = 0.008), *bla*_CTX-M_ (29.3% vs. 17.6%; p = 0.025), and *bla*_OXA_ (14.3% vs. 6.4%; p = 0.037). Total ESBL carriage for bacteremia isolates was also significantly higher than for urine isolates (59.3% vs. 29.6%; p<0.001).

Multilocus sequence types were determined for all *E. coli* isolates. A total of 63 STs were found among the urine isolates (Figure, panel A); the highest prevalence was ST73 (n = 16, 12.8%), followed by ST131 (n = 9, 7.2%), ST69 (n = 9, 7.2%), ST95 (n = 6, 4.8%), ST404 (n = 6, 4.8%), ST127 (n = 4, 3.2%), ST141 (n = 4, 3.2%), and ST10 (n = 3, 2.4%). Prevalence patterns of STs among bacteremia *E. coli* isolates were noticeably different (Figure, panel B). Three main STs were obtained. ST131 dominated (n = 30, 21.43%) and was significantly higher in prevalence than for the urine isolates (p<0.001). ST73 (n = 24, 17.14%) and ST95 (n = 13, 9.29%) were the other 2 primary STs found. The 8 most prevalent STs in the bacteremia isolates represented 59.29% of the total population, whereas the 8 most prevalent STs in the urine isolates represented 45.6% of the total population. This finding is suggestive of selection of a smaller number of dominant STs in bacteremia.

**Figure:**
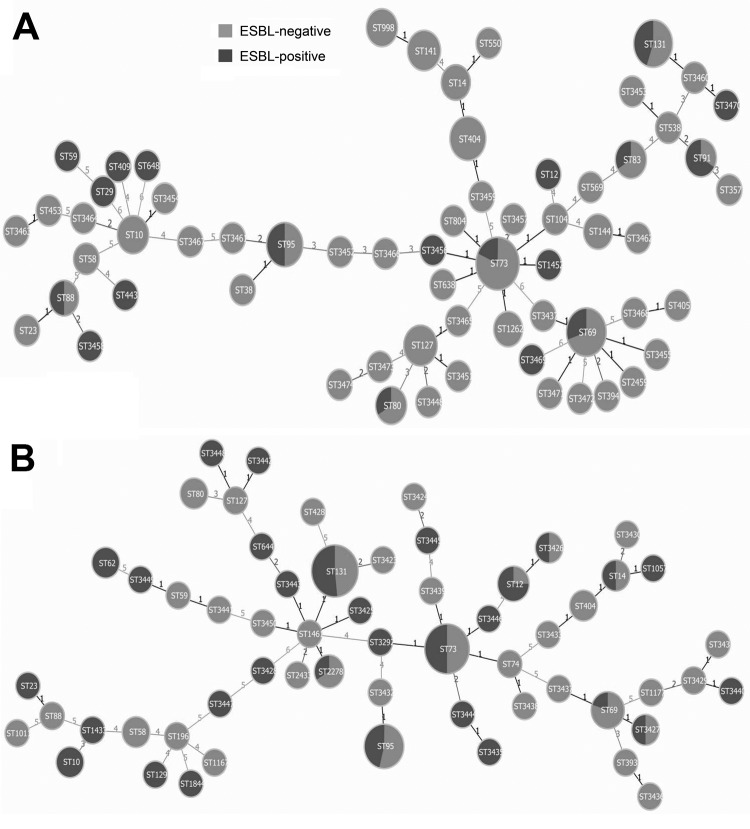
Minimum-spanning trees showing carriage of extended-spectrum β-lactamases (ESBL) in *Escherichia coli* isolates from urine samples (A) and samples from patients with bacteremia (B). Each circle represents 1 sequence type (ST), and the size of the circle reflects the number of isolates belonging to this particular ST within the bacteria group. Lines between the circles represent how different their allelic profiles are; a line labeled 1 means the linked STs differ in >1 of the 7 alleles, which is named a single locus variant (SLV). A cluster of STs linked by SLVs is a clonal complex. Nineteen (30.16%) of 63 STs found among the urine isolates were ESBL positive, in comparison to 30 (51.72%) of 58 for the bacteremia isolates.

ESBL carriage was mapped onto minimum-spanning trees for the 2 isolate groups. ESBL carriage among urine isolates was focused on a small number of STs; 19 (30.16%) of the 63 STs contained ESBL-positive isolates (Figure, panel A). The predominant ST73 group contained 18.75% ESBL-positive isolates; the other predominant STs exhibited ESBL-positive isolates at the following levels: ST131 (44.44%), ST69 (33.33%), ST95 (50%), and ST10 (0%). In contrast, 30 (51.72%) of the 58 STs among bacteremia isolates contained ESBL-positive isolates, significantly higher than for the urine isolates (p = 0.016). At the ST level, predominant STs had higher ESBL carriage in the bacteremia isolates than in the urine isolates: ST131 (50%), ST73 (50%), ST12 (75%), ST10 (100%), ST14 (50%), ST2278 (33.33%). ST95 (46.15%) and ST69 (20%) showed comparable levels. These results suggest that ESBL drug resistance is selecting for dominant ExPEC bacteremia strains.

To investigate whether the differences in ST observations between bacteremia and urine isolates could be attributable to differences in virulence genes, VAGs of all isolates were screened by multiplex PCR. VAGs were found equally distributed across the 2 populations, with no statistically significant difference (p = 0.675). Comparison of serum resistance levels between urine and blood isolates also showed no phenotypic differences.

In conclusion, we found high levels of ESBL carriage and multidrug resistance in ExPEC isolates that cause bacteremia. A comparison with urine isolates provided evidence that ESBL-mediated drug resistance appears to be the selective pressure in the emergence of dominant STs in bacteremia. Future research should focus on identifying if prolonged antimicrobial drug treatment in bacteremia patients is leading to this selection.
